# Influence of Magnetic Field and Temperature on Rheological Behavior of Magnetorheological Gel

**DOI:** 10.3390/ma15228070

**Published:** 2022-11-15

**Authors:** Min Sun, Xiangdong Li, Zhou Zhou, Ran Deng, Xu Chen, Jiong Wang, Runsong Mao

**Affiliations:** 1 Jiangsu Special Equipment Safety Supervision and Inspection Institute, Nanjing 210002, China; 2 School of Mechanical Engineering, Nanjing University of Science and Technology, Nanjing 210094, China

**Keywords:** magnetorheological gel, dynamic yield stress, normal stress, relative MR effect

## Abstract

In this paper, the effect of temperature on rheological properties of magnetorheological (MR) gel is investigated under rotational steady shear and oscillatory dynamic shear. A kind of fluid-like MR gel (MRG) was firstly synthesized by mixing carbonyl iron powder (CIP) with polymer matrix. Then, the relationship between yield stress, normal stress of MRG and shear rate under six temperatures and four magnetic field strengths were studied by rotational shear experiments. The results demonstrate that the dependence of shear stress on temperature displays an opposite tendency in comparison with that of normal stress on temperature. Moreover, maximum yield stress, one of the most important parameter of MR materials, decreases with the increment of temperature. Under oscillatory dynamic shear test, storage and loss moduli and normal stress of MRG all increase with temperature when a magnetic field is applied, which presents a contrary trend in the absence of a magnetic field. Related mechanisms about the alternation of microstructures of MRG were proposed to explain the above-mentioned phenomena. This paper is helpful in fabricating semi-active engineering devices using MR materials as a medium.

## 1. Introduction

Prepared by blending iron particles with polymer matrix, magnetorheological (MR) gel whose rheological properties could be adjusted by an external magnetic field is a new sort of magnetic-sensitive intelligent material [1,2]. The randomly dispersed particles in MR gel (MRG) will aggregate together to form chain-like or column-like structures when a magnetic field is applied. Conversely, once the magnetic field is removed, these particles will rearrange to restore to their original state because of the weak restriction of polymer matrix to them [3]. Furthermore, it is due to existence of the polymer matrix without sufficient crosslinking that MRG could grapple with the settlement issue in traditional MR fluid (MRF) because of the density mismatch between its oil matrix and particle, and low MR effect of conventional MR elastomer (MRE) owing to the strong constraint of its rubber matrix to particles [4]. Therefore, it is hugely promising for MRG to be employed in MR dampers [5], clutches [6] and absorbers [7,8], etc.

Publications about MRG by far concentrate on effects of different excitation inputs on rheological response, such as shear stress, storage modulus, apparent viscosity, loss modulus, under shear mode. Sousa et al. [9] characterized MR effect of MRG with different kinds of polymer matrixes and particles by creep-recovery test and angular frequency sweep test and found that influence of a gelatinous matrix on the MR effect of MRG is greater than that of particle size while particle distribution in matrix could also highly affect rheological properties of MRG. Moreover, fabricating spherical $C_{0}Fe_{2}O_{4}$ nanoparticles by sol-gel hydrothermal method, Hajalilou et al. [10] added them into a micron-sized carbonyl iron (CI)-based MRF. Compared with the commonly used CI-based MRF, $C_{0}Fe_{2}O_{4}$-CI-based MRF possessed larger shear yield stress, apparent viscosity and MR effect because of the higher saturation magnetization of smaller particles. Yu et al. [11] synthesized a brand new MRG by coating CI particles with dendritic-like Co particles whose saturation magnetization is much higher than that of CI particles. When a magnetic flux density of 300 mT is applied, the maximum dynamic yield stress of this MRG is 10.443 kPa, which is 1.53 times higher than that of pure CI-based MRG. Plachy et al. [12] oxidized carbonyl iron (CI) particles thermally at 500 °C to investigate the impact of corrosion process of CI particles on MR performance of MR suspensions. For expanding the application of MR materials on biomechanical or biomedical fields, Cvek et al. [13] fabricated a novel poly (2-oxazoline)-based magnetic hydrogel and characterized its unique performance and cytotoxicity. A particle-level simulation method was adopted by Gong et al. [14] to investigate the normal stress of solid-like MRG under large oscillatory amplitude shear where it presents three different changing trends: keeping constant when the shear strain is below 0.1%, improving with the increase of strain when the strain is between 0.1% and 0.8%, and decreasing with the growth of strain when the stain in the interval of 0.8% and 100%. However, normal stress of MRG under quasi-static shear condition displays a sudden growth, following with a fast reduction with the increasing of shear strain, and a constant level after a critical shear strain when a magnetic field is applied. This is possibly because MRG would appear different rheological properties under different shear conditions [15]. Furthermore, Kim et al. [16] employed MRG as the controllable medium to design an adaptive tunable vibration absorber that could adjust the frequency of the primary system from 56 Hz to 67 Hz by control the stiffness of MRG. Simultaneously, they used a proportion-integral-differential (PID) controller to realize the desired stiffness of MRG by controlling the required magnetic field.

Apart from the above-mentioned factors, temperature is also an important controlling parameter to change the rheological performance of MRG because the polymer matrix is highly temperature-dependent. In addition, frequent shear, vibration and input current, etc. all would result in the variations of temperature of MRG, thus changing its property. Therefore, exploration of how temperature affects rheological behavior of MRG is urgent because the property of MRG is highly relevant to the performance of an MR device. However, investigations about the influence of temperature on the shear response of MRG by far are rare. Experiments conducted by Yu et al. [17] demonstrated that temperature could adjust the normal force of MRG under high magnetic fields (higher than 0.6 T), whereas nearly has no effect on it under low magnetic fields. Moreover, Xu et al. [18] found that the creep stain of solid-like MRG decreases with the increase of temperature without a magnetic field while presents a contrary tendency under a 930 mT magnetic field flux density. Fortunately, there existed several references about the effect of temperature on the rheological properties of other MR materials (as shown in Table 1), and these references could provide some guidance for us to study the temperature-dependency of MRG.

In this paper, the rheological properties of MRG under different temperatures are investigated. Some MRG samples with constant 70% weight fraction of CI powders (CIPs) were firstly fabricated. Then, experiments about variation of shear stress with shear rate under different magnetic fields and temperatures were conducted. Except for the rotational shear tests, influence of temperature on the storage modulus, loss modulus and normal stress of MRG under oscillatory shear were tested. Furthermore, relevant mechanisms from the perspective of internal structures of MRG are discussed for explaining the temperature-dependency. The main highlights of this paper could be summarized as follows: the first one is that a systematic investigation on the temperature-dependent rheological property of MRG is presented. Previous studies regarding temperature-dependent property of MRG basically concentrated on modulus and static normal stress, and with limited length. Secondly, dependence of normal stress of MRG on temperature under rotational and oscillatory shear is studied. Although there already exist many papers reporting the temperature-dependent properties of MR materials, discussion about the influence of temperature on normal stress under steady and dynamic conditions is rare. Last, relevant mechanism are proposed to explain the coupling effect between temperature and magnetic field. This paper elaborates the coupling effect of various external excitations, i.e., temperature, magnetic field, and shear strain, on the evolution of particle microstructures and polymer matrix.

## 2. Materials and Methods

### 2.1. Preparation of MRG Samples

**Figure 1:**
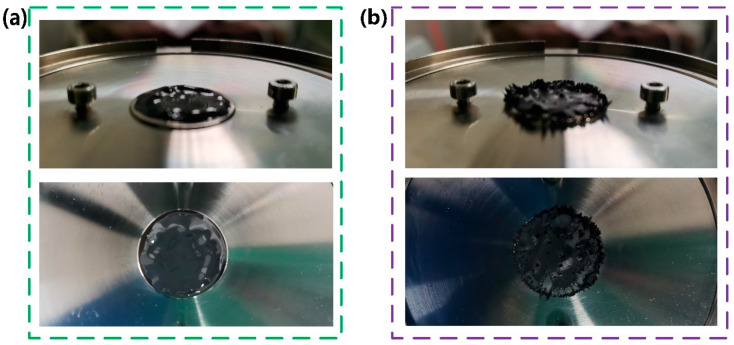
Photos of MRG-70 under 0 kA/m (**a**) and 293 kA/m (**b**) magnetic field strength.

In this paper, carbonyl iron powder (CIPs, type CN, BASF, Germany with an average particle size of approximately 6 μm) as a dispersed phase and polymer matrix as a continuous phase were chosen to prepare our fluid-like MRG samples. For polymer matrix, polypropylene glycol (PPG-2000, $M_{n}$ = 2000, Sigma-Aldrich (Shanghai) Trading Co., Ltd., Shanghai, China) and toluene diisocyanate (TDI, 2,4- ≈ 80%, 2,6- ≈ 20%, Tokyo Chemical Industry Co., Ltd., Japan) were selected as two main synthetic ingredients which are blended with a mechanical stirrer under 80 °C. Next, dipropylene glycol (SOL, Sigma-Aldrich (Shanghai) Trading Co., Ltd., Shanghai, China) as chain extender and moderate catalyst were added into the reaction, thus finishing the fabrication of polymer matrix. More details about the synthesis of polymer matrix, such as the exact amount of the reagents, could be found in our previous publications [23,24,25]. Finally, MRG samples with 70% CIPs contents were prepared by stirring CIPs and polymer matrix for one hour at 1200 rpm, and named as MRG-70. It can be observed from Figure 1a that MRG behaves like a viscoelastic fluid where magnetic particles distribute evenly without a magnetic field. However, the magnetic particles would aggregate together to form chain-like structures due to magnetic attractive force between particles when a magnetic field is applied [15].

### 2.2. Temperature Experiments

A commercial advanced rheometer (Type MCR 302, Anton Paar Co., Craz, Austria) is used to measure rheological properties of MRG under different magnetic fields and temperatures. MRG samples were placed in the lower plate of the rheometer, and the upper plate could apply excitation signals, such as shear rate and shear strain, defined by customers. The diameter of plates is 20 mm and the distance between them is set for 1 mm. Moreover, the magnetorheological module (Type MRD 180, Anton Paar Co., Craz, Austria) and temperature accessory (Type F25, Julabo Technology Co., Seelbach, Germany) could change the applied current from 0 A to 5 A (i.e., from 0 kA/m to 866 kA/m) and the temperatures from 10 °C to 70 °C, respectively.

**Figure 2:**
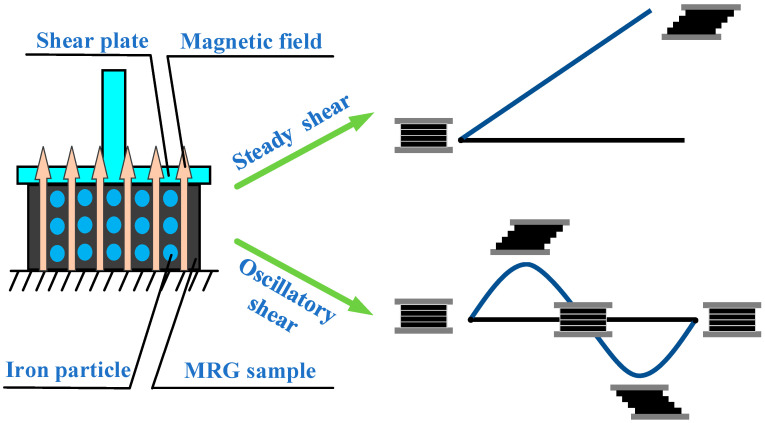
Schematic diagram of test principle.

As shown in Figure 2, two kinds of shear test, rotational steady shear and oscillatory dynamic shear, are employed to investigate the rheological properties of MRG under different temperatures. The upper plate applies a steady shear with shear rate varying from 0.01 $s^{-1}$ to 100 $s^{-1}$ to MRG samples, while a dynamic shear with shear strain ranging from 0.01% to 10%. Then the data about shear stress, storage modulus, loss modulus and normal stress of MRG could be collected by the dynamic signal analyzer embedded in rheometer. Moreover, the test environmental temperatures are set as 10 °C, 25 °C, 40 °C, 50 °C, 60 °C and 70 °C while magnetic field strengths are set as 0 kA/m, 96 kA/m, 194 kA/m and 293 kA/m. Moreover, for oscillatory shear, the frequency is set as 5 Hz. It is necessary to mention that every test was repeated three times to ensure the accuracy and repeatability of data and that MRG samples were stirred for half an hour at 1200 rpm before every experiment.

## 3. Results and Discussion

### 3.1. Influence of Temperature on Shear Stress

**Figure 3:**
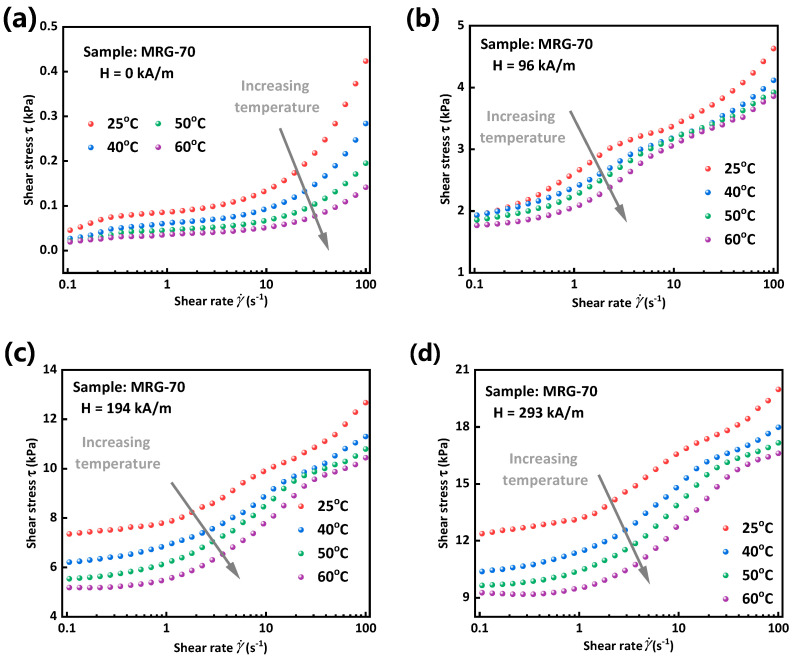
Shear stress as a function of shear rate under different temperatures and magnetic field strengths. 0 kA/m (**a**), 96 kA/m (**b**), 194 kA/m (**c**) and 293 kA/m (**d**).

It can be seen from Figure 3a that the shear stress of MRG increases linearly with shear rate in the absence of a magnetic field, no matter how large the environmental temperature is. This indicates that MRG behaves like a Newtonian fluid when magnetic particles disperse randomly in matrix. However, from Figure 3b–d, MRG will present Bingham plastic fluid behavior [26] under a magnetic field where shear stress firstly appears a sharp increase and then a slow growth with a nearly constant slope. Moreover, similar to MRF, shear stress and apparent viscosity, defined as the slope of shear stress-shear rate, of MRG both decrease with the increase of temperature regardless of the value of magnetic field strength. Increasing of apparent viscosity with the decline of temperature is possibly because relatively lower temperature ‘freezes’ the molecular chains of polymer matrix, thus letting the movement of matrix harder [27]. Simultaneously, friction between particle chains and the frozen matrix would also become larger due to the decrease of temperature, which results in much larger shear stress needed to make MRG yield. Therefore, shear stress and apparent viscosity of MRG increase with the decrease of environmental temperature.

**Figure 4:**
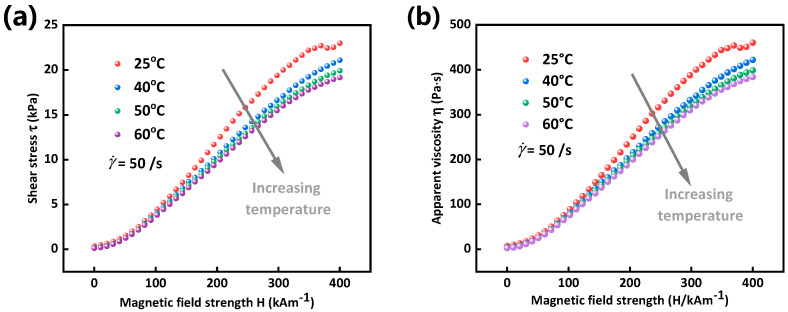
Shear stress (**a**) and apparent viscosity (**b**) as a function of magnetic field strength under different temperatures.

Apart from the shear rate-dependency of shear stress of MRG, magnetic field strength-dependency of shear stress is also worthy to be investigated since magnetic field is the most important factor influencing MR materials. Therefore, a magnetic field strength sweep test was conducted under constant shear rate of 50 $s^{-1}$. It can be seen from Figure 4 that both shear stress and apparent viscosity of MRG increase with the increase of magnetic field strength and decrease with the increment of temperature, which are identical to the results presented in Figure 3. When a magnetic field is applied, magnetic interaction between particles contributes to the formation of chain-like microstructures of MRG. The higher the magnetic field strength is, the larger the particle-particle magnetic interaction becomes. Thus, shear stress of MRG increase with the increase of magnetic field. It is necessary to note here that the increasing speed of shear stress, i.e., the slope of shear stress-magnetic field strength, presents a gradual growth and then a progressive decline. This is possibly related to the magnetization property of the chosen magnetic particle that will gradually reach magnetization saturation with the increase of magnetic field [28]. Another interesting phenomenon is that temperature almost has no effect on shear stress and apparent viscosity of MRG under low magnetic field, most notably below 150 kA/m magnetic field strength. That is to say, temperature-dependency of MRG is higher under higher magnetic field than that under lower magnetic field strength. When magnetic field strength is relatively lower, magnetic field, as the most influential element, will drive the magnetic particles to form more steady chain structures at this time when revolution of microstructures is mainly decided by magnetic field instead temperature. Thus, the influence of temperature is relatively less under lower magnetic field strength. However, magnetic interaction between particles will gradually tend to be stable with the increasing of magnetic field strength, and hardly could be changed again. Temperature, as a controlling factor, will play a role in changing the property of polymer matrix at this moment, which influences the rheological property of MRG. Therefore, influence of temperature on shear stress and viscosity is more evident under higher magnetic field strength.

### 3.2. Influence of Temperature on Maximum Yield Stress

**Figure 5:**
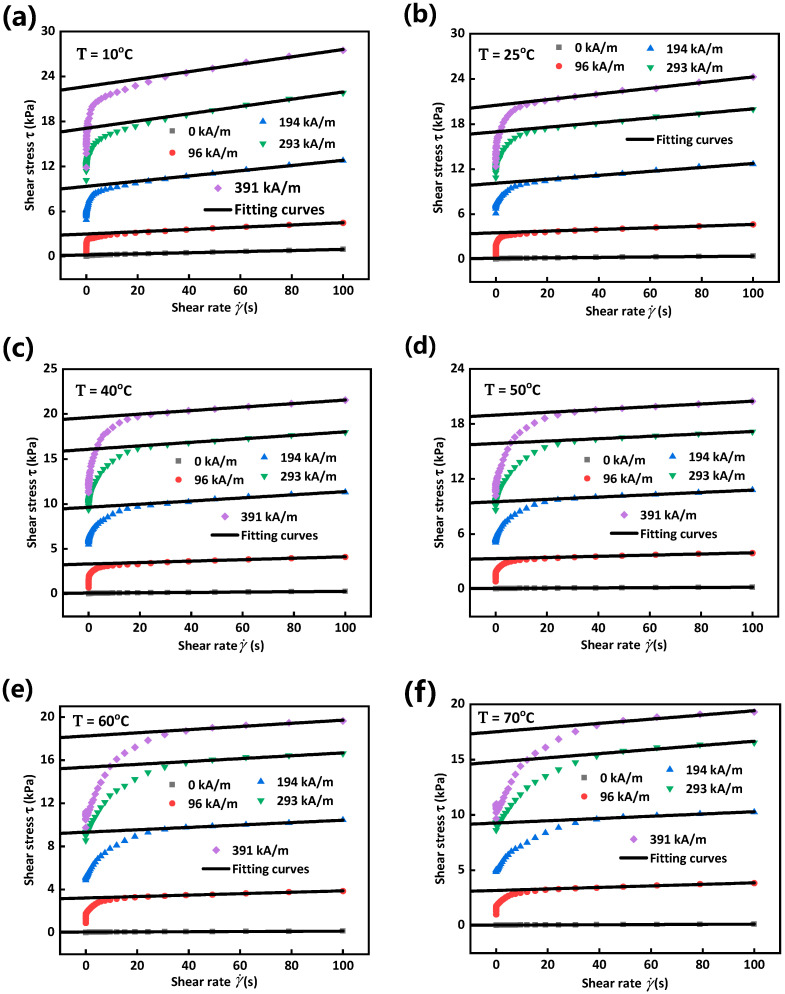
Fitting curves of maximum yield stress of MRG under different magnetic field strengths and temperatures. 10 °C (**a**), 25 °C (**b**), 40 °C (**c**), 50 °C (**d**), 60 °C (**e**) and 70 °C (**f**).

Figure 5 displays the effect of temperature on maximum yield stress of MRG which is one of the most significant parameters in designing MR device. It is necessary to note here that the yield stress presented in Figure 5 is the so-called Bingham yield stress. Normally, two kinds of different methods are adopted to characterize the yield stress [29,30]: static yield stress which is determined as the stress representing the beginning of flow in log-log coordinate of shear stress versus shear rate, and Bingham yield stress which is determined as the stress achieved by fitting stress–rate curves in lin–lin coordinates with Bingham plastic model. Although there existed many other approaches that could be used to attain yield stress, the above-mentioned two approaches are most commonly used. Moreover, in this paper, Bingham yield stress is chosen as our focus. The expression of the Bingham plastic model is [31,32]:

(1) $$\left\{\begin{array}{ll} \tau =\tau_{y}+\eta \cdot \dot{\gamma } & \tau >\tau_{y}\\ \dot{\gamma }=0 & \tau <\tau_{y} \end{array}\right.$$

**Figure 6:**
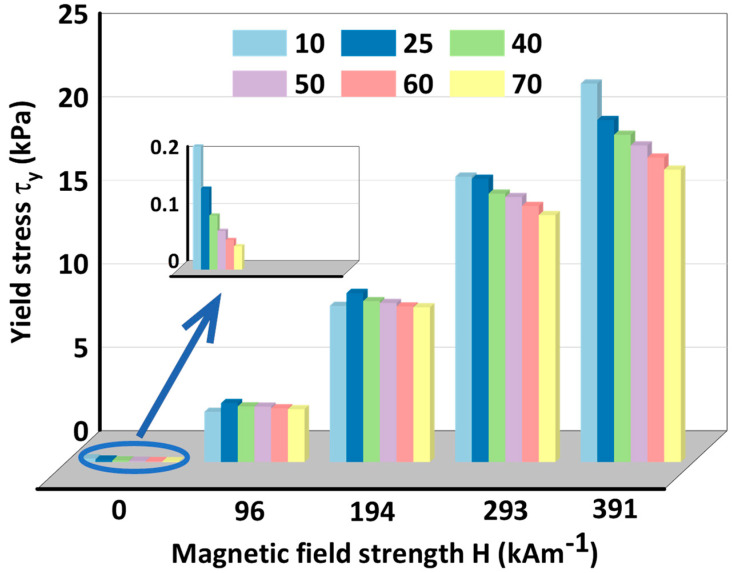
Maximum yield stress of MRG as a function of temperature under different magnetic field strengths.

Where τ, $\tau_{y}$, η and $\dot{\gamma }$ denote shear stress, yield stress, plastic viscosity and shear rate, respectively. The fitting curves and magnitudes of maximum yield stress under six different temperatures and five magnetic field strengths are presented in Figure 5 and Figure 6. It can be seen from Figure 6 that yield stress of MRG basically decreases with the increase of temperature and the decreased value drops with the increment of magnetic field strength.

### 3.3. Influence of Temperature on Storage Modulus

**Figure 7:**
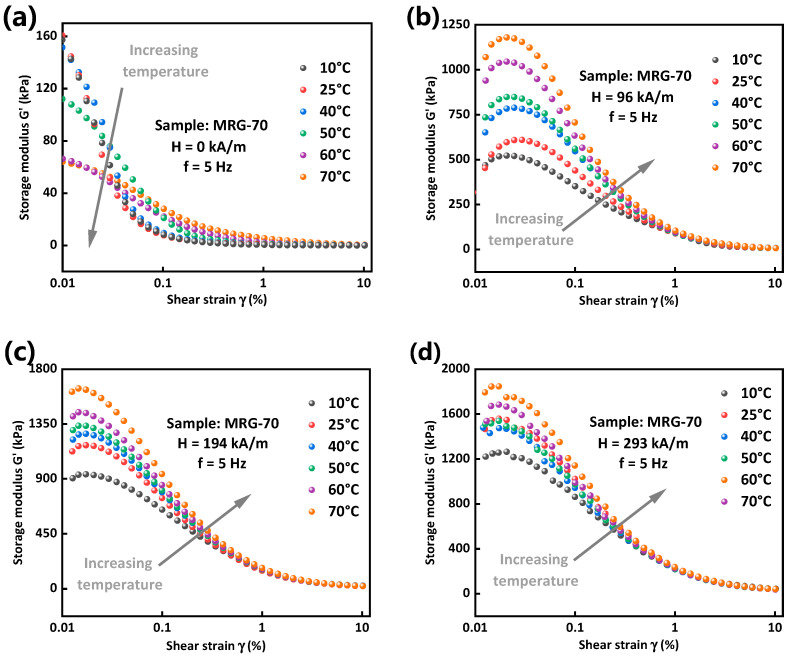
Storage modulus of MRG as a function of shear strain under different temperatures. 0 kA/m (**a**), 96 kA/m (**b**), 194 kA/m (**c**) and 293 kA/m (**d**).

Storage modulus typically represents the ability of a viscoelastic material to store the deformation energy [33]. A strain amplitude sweep experiment was conducted to investigate the storage modulus of MRG under six different temperatures and four different magnetic field strengths, and the results are presented in Figure 7. In the absence of a magnetic field, storage modulus of MRG decreases with the increase of shear strain and finally levels off. Moreover, temperature has a great effect on the storage modulus of MRG under small strain amplitude, increasing from 64 kPa under 70 °C to 157 kPa under 10 °C when shear strain amplitude is 0.01%. However, this effect will decrease under high strain amplitude where the curves of storage modulus under different temperatures nearly overlap with each other. When a magnetic field is applied, storage modulus of MRG increases slightly, then decreases sharply and finally reaches a plateau with the increase of shear strain. Interestingly, it can be seen from Figure 7b–d that the difference between storage modulus under different temperatures gradually decreases with the increase of magnetic field strength, which indicates that a magnetic field could weaken the effect of temperature on ability of MRG to store the deformation energy. For example, under 293 kA/m magnetic field strength, the storage modulus of MRG increases from 1221 kPa under 10 °C to 1793 kPa under 70 °C when the shear strain amplitude is 0.01%. The increase ratio of storage modulus is 47% which is much smaller than 145% under 0 kA/m magnetic field strength.

### 3.4. Influence of Temperature on Loss Modulus

**Figure 8:**
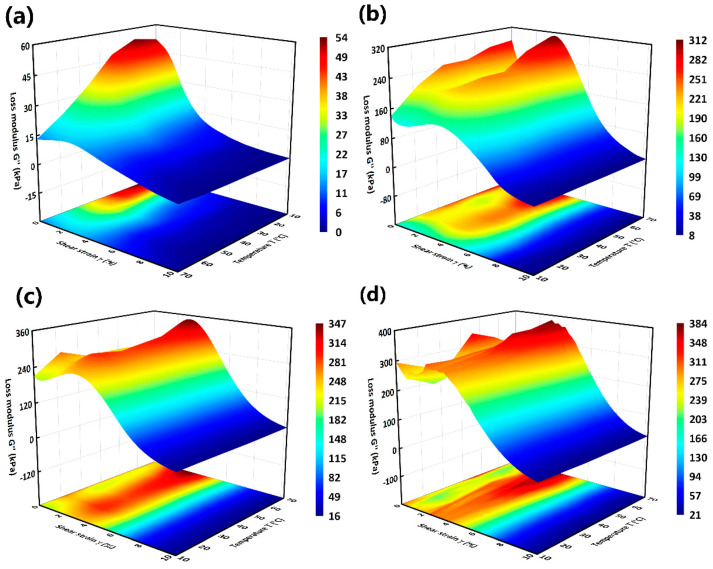
Loss modulus of MRG as a function of shear strain under different temperatures. 0 kA/m (**a**), 96 kA/m (**b**), 194 kA/m (**c**) and 293 kA/m (**d**).

Loss modulus is another parameter that is closely related to the ability of a viscoelastic material to dissipate the deformation energy [28]. Figure 8 displays the dependency of strain amplitude on loss modulus of MRG under different temperatures and magnetic field strengths. Loss modulus of MRG decreases with the increase of shear strain (as shown in Figure 8a) without a magnetic field, while shows slight decline, then a growth and finally a dramatic decline with the increment of shear strain in the presence of a magnetic field. Similar with storage modulus, loss modulus of MRG is almost independent with temperature under high strain amplitudes, as the dark blue of the 3D surface presented. Moreover, the higher the temperature is, the larger the loss modulus of MRG is, which means that better ability of MRG to dissipate the deformation energy happens at higher temperature when a magnetic field is applied. This is possibly because higher temperature makes the movements of magnetic particles and molecular chains in matrix more intense, contributing to more interfacial slipping between particle chains as well as particles and matrix [34]. Therefore, in the presence of a magnetic field, more deformation energy is dissipated under higher temperature. Furthermore, loss modulus of MRG increases with magnetic field strength, resulting from the more complicated particle-chain structures in MRG under higher magnetic field which more energy is needed to destroy [35].

### 3.5. Influence of Temperature on Normal Stress

**Figure 9:**
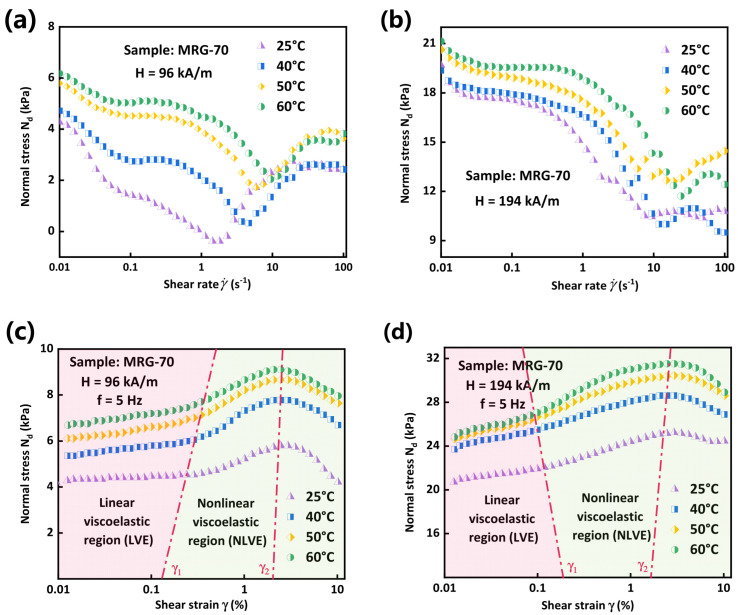
Normal stress of MRG as function of shear rate under 96 kA/m (**a**) and 194 kA/m (**b**), and shear strain under 96 kA/m (**c**) and 194 kA/m (**d**).

Besides, variations of normal stress with shear strain and shear rate under different temperatures and magnetic field strengths are also presented in Figure 9. The normal stress could be calculated by [36] $N_{d}=8F_{n}/\pi d^{2}$, where $F_{n}$, d and $N_{d}$ are normal force collected by MCR 302, diameter of the plate of MCR 302 and normal stress of MRG. It is necessary to note here that normal stress in the absence of a magnetic field is nearly negligible [15], thus is not presented here. It can be seen from Figure 9 that, regardless of the value of temperature, normal stress displays a peak with the increase of shear strain while a trough with the increase of shear rate. Moreover, normal stress increases with the increment of temperature under shear rate sweep test, contrary to the increasing trend of shear stress. However, variation tendency of normal stress is much more complicated than that of shear stress. Furthermore, the variation of normal stress with shear strain could divide into two regions: linear viscoelastic region (LVE, the red region) where normal stress keeps almost constant, and nonlinear viscoelastic region (NVLE, the green region) where normal stress increases to a peak and then decreases with shear strain [37]. When the shear strain is relatively small ($\gamma <\gamma_{1}$), the stiff chain-like structures forming due to a magnetic field would not be destroyed by the strain, which leads to the nearly constant level of normal stress at the initial stage. With the increase of shear strain, single particle-chain attractive with each other to form more complicated and stiffer column-like or net-like microstructures [38] in MRG to resist the continuous increasing deformation. Therefore, the normal stress of MRG increases with the increase of shear strain. When the shear strain amplitude exceeds $\gamma_{2}$, deformation is large enough to break the structures in MRG, thus resulting in the decrease of normal stress of MRG. Moreover, it is necessary to note here that the $\gamma_{1}$ and $\gamma_{2}$ are only two qualitive concepts rather two quantitative values.

## 4. Conclusions

In this paper, a fluid-like MRG was fabricated by dispersing 70% weight fraction of CIP into polymer matrix, and influence of temperature on the rheological properties of MRG was studied by rotational and oscillatory shear experiments. It is found that shear stress of MRG nonlinearly decreases with the increase of environmental temperature under shear rate sweep test. Moreover, MRG behaves like a Newtonian fluid in the absence of a magnetic field while a Bingham plastic fluid in the presence of a magnetic field. However, variation of normal stress with temperature presents an opposite tendency, i.e., increases with the temperature, under the rotational shear test. Furthermore, influence of magnetic field on maximum yield stress of MRG increases with temperature, whereas the effect of temperature on maximum yield stress of MRG is more preeminent at 0 kA/m magnetic field strength. Under oscillatory shear experiments, storage modulus, loss modulus and normal stress of MRG all improve with the increment of temperature when a magnetic field is applied. Furthermore, the normal stress could be divided into three stages: a steady value, then an increasing trend and finally a gradual decline, which possibly results from the complex transformation of the internal microstructures of MRG. The improvement of storage and loss moduli, in the presence of a magnetic field, with temperature indicates that MRG could store more and dissipate more deformation energy under relatively high temperature. This is possibly because the migration of magnetic particles and molecular chains in matrix becomes more intense under higher temperature, thus making the particle chains stiffer and the interfacial slipping between particles and matrix more evident. This paper could provide some guidance for designing practical device employing MRG.

## Figures and Tables

**Table 1:** References that studied the influence of temperature on rheological properties of MR materials.

Materials	Research Contents	Range of Temperature	Range of Magnetic Field
MRF [19]	Shear stress Storage modulus Loss modulus	20 °C~55 °C	0.1 T~0.5 T
MRF [20]	Shear stress Yield stress	25 °C~40 °C	0 mT~540 mT
MRE [21]	Storage modulus Loss modulus	25 °C~60 °C	0 mT~500 mT
MRE [22]	Storage modulus Stress relaxation behavior	20 °C~50 °C	0 kA/m~490 kA/m
MRG [18]	Creep and recovery behavior	25 °C~75 °C	0 mT and 900 mT
MRG [17]	Static normal force	20 °C~80 °C	0 T~0.8 T

## Data Availability

The data could be attained by contacting the corresponding authors.

## References

[1] Meharthaj H., Srinivasan S.M., Arockiarajan A. (2020). Creep behavior of magnetorheological gels. Mech. Adv. Mater. Struct..

[2] Pang H.M., Pei L., Xu J.Q., Cao S.S., Wang Y., Gong X.L. (2020). Magnetically tunable adhesion of composite pads with magnetorheological polymer gel cores. Compos. Sci. Technol..

[3] Xu Y.G., Liu T.X., Liao G.J., Lubineau G. (2017). Magneto-dependent stress relaxation of magnetorheological gels. Smart Mater. Struct..

[4] Xu Y.G., Gong X.L., Xuan S.H., Zhang W., Fan Y.C. (2011). A high-performance magnetorheological material: Preparation, characterization and magnetic-mechanic coupling properties. Soft Matter.

[5] Yang Y., Xu Z.-D., Guo Y.-Q., Sun C.-L., Zhang J. (2021). Performance tests and microstructure-based sigmoid model for a three-coil magnetorheological damper. Struct. Control Health Monit..

[6] Yadmellat P., Kermani M.R. (2014). Adaptive Modeling of a Magnetorheological Clutch. IEEE/ASME Trans. Mechatron..

[7] Park J.-E., Lee J., Kim Y.-K. (2021). Design of model-free reinforcement learning control for tunable vibration absorber system based on magnetorheological elastomer. Smart Mater. Struct..

[8] Xuan Bao N., Komatsuzaki T., Hoa Thi T. (2020). Novel semiactive suspension using a magnetorheological elastomer (MRE)-based absorber and adaptive neural network controller for systems with input constraints. Mech. Sci..

[9] Gomes de Sousa S.R., dos Santos M.P., Faria Bombard A.J. (2019). Magnetorheological gel based on mineral oil and polystyrene-b-poly(ethene-co-butadiene)-b-polystyrene. Smart Mater. Struct..

[10] Hajalilou A., Mazlan S.A., Abbasi M., Lavvafi H. (2016). Fabrication of spherical CoFe2O4 nanoparticles via sol-gel and hydrothermal methods and investigation of their magnetorheological characteristics. Rsc. Adv..

[11] Wang L., Yu M., Fu J., Qi S. (2018). Investigation on the effects of doped dendritic Co particles on rheological property of magnetorheological gel. Smart Mater. Struct..

[12] Plachy T., Kutalkova E., Sedlacik M., Vesel A., Masar M., Kuritka I. (2018). Impact of corrosion process of carbonyl iron particles on magnetorheological behavior of their suspensions. J. Ind. Eng. Chem..

[13] Cvek M., Zahoranova A., Mrlik M., Sramkova P., Minarik A., Sedlacik M. (2020). Poly(2-oxazoline)-based magnetic hydrogels: Synthesis, performance and cytotoxicity. Colloids Surf. B Biointerfaces.

[14] Pang H., Pei L., Sun C., Gong X. (2018). Normal stress in magnetorheological polymer gel under large amplitude oscillatory shear. J. Rheol..

[15] Mao R., Ye X., Wang H., Zhang G., Wang J. (2021). Magneto-Induced Normal Stress of Magnetorheological Gel under Quasi-Statically Monotonic and Periodically Cyclic Loading. Front. Mater..

[16] Kim H.K., Kim H.S., Kim Y.-K. (2017). Stiffness control of magnetorheological gels for adaptive tunable vibration absorber. Smart Mater. Struct..

[17] Ju B.X., Yu M., Fu J., Zheng X., Liu S.Z. (2013). Magnetic Field-Dependent Normal Force of Magnetorheological Gel. Ind. Eng. Chem. Res..

[18] Xu Y.G., Gong X.L., Xuan S.H., Li X.F., Qin L.J., Jiang W.Q. (2012). Creep and recovery behaviors of magnetorheological plastomer and its magnetic-dependent properties. Soft Matter.

[19] Arief I., Mukhopadhyay P.K. (2017). Yielding behavior and temperature-induced on-field oscillatory rheological studies in a novel MR suspension containing polymer-capped Fe3Ni alloy microspheres. J. Magn. Magn. Mater..

[20] Bahiuddin I., Mazlan S.A., Shapiai I., Imaduddin F., Choi S.-B. (2018). Constitutive models of magnetorheological fluids having temperature-dependent prediction parameter. Smart Mater. Struct..

[21] Wan Y., Xiong Y., Zhang S. (2018). Temperature dependent dynamic mechanical properties of Magnetorheological elastomers: Experiment and modeling. Compos. Struct..

[22] Wen Q., Shen L., Li J., Xuan S., Li Z., Fan X., Li B., Gong X. (2020). Temperature dependent magneto-mechanical properties of magnetorheological elastomers. J. Magn. Magn. Mater..

[23] Mao R.S., Wang H.X., Zhang G., Ye X.D., Wang J. (2020). Magneto-induced rheological properties of magnetorheological gel under quasi-static shear with large deformation. RSC Adv..

[24] Mao R., Zhang G., Wang H., Wang J. (2022). Temperature-dependent dynamic properties of magnetorheological gel composite: Experiment and modeling. Smart Mater. Struct..

[25] Mao R., Wang X., Cai S., Zhang G., Wang J. (2022). Strain dependent magneto-mechanical property of magnetorheological gel composite: Rheological measurement and model establishment. Compos. Sci. Technol..

[26] de Vicente J., Klingenberg D.J., Hidalgo-Alvarez R. (2011). Magnetorheological fluids: A review. Soft Matter.

[27] Hemmatian M., Sedaghati R., Rakheja S. (2020). Temperature dependency of magnetorheological fluids’ properties under varying strain amplitude and rate. J. Magn. Magn. Mater..

[28] Wang H., Li Y., Zhang G., Wang J. (2019). Effect of temperature on rheological properties of lithium-based magnetorheological grease. Smart Mater. Struct..

[29] de Vicente J., Berli C.L.A. (2013). Aging, rejuvenation, and thixotropy in yielding magnetorheological fluids. Rheol. Acta.

[30] Li Z., Li D., Hao D., Cheng Y. (2017). Study on the creep and recovery behaviors of ferrofluids. Smart Mater. Struct..

[31] Chen K., Yu X., Wang H., Zheng H., Zhang G., Wu R. (2021). Modeling of a bingham model of a magnetorheological damper considering stochastic uncertainties in their geometric variables. J. Theor. Appl. Mech..

[32] Yang Y., Yu P., Zhu S., Zhao J. (2015). Nonlinear Bingham Model of a Magnetorheological Shock Absorber. Proceedings of the 2015 International Conference on Industrial Technology and Management Science.

[33] Gong X.L., Xu Y.G., Xuan S.H., Guo C.Y., Zong L.H., Jiang W.Q. (2012). The investigation on the nonlinearity of plasticine-like magnetorheological material under oscillatory shear rheometry. J. Rheol..

[34] Qi S., Yu M., Fu J., Li P.D., Zhu M. (2016). Creep and recovery behaviors of magnetorheological elastomer based on polyurethane/epoxy resin IPNs matrix. Smart Mater. Struct..

[35] Xu Y.G., Liu T.X., Wan Q., Gong X.L., Xuan S.H. (2015). The energy dissipation behaviors of magneto-sensitive polymer gel under cyclic shear loading. Mater. Lett..

[36] Jiang J., Tian Y., Ren D., Meng Y. (2011). An experimental study on the normal stress of magnetorheological fluids. Smart Mater. Struct..

[37] Guo C., Gong X., Xuan S., Zong L., Peng C. (2012). Normal forces of magnetorheological fluids under oscillatory shear. J. Magn. Magn. Mater..

[38] Liu T.X., Xu Y.G., Gong X.L., Pang H.M., Xuan S.H. (2013). Magneto-induced normal stress of magnetorheological plastomer. AIP Adv..

